# Abnormalities in resting-state EEG microstates are a vulnerability marker of migraine

**DOI:** 10.1186/s10194-022-01414-y

**Published:** 2022-04-05

**Authors:** Yansong Li, Guoliang Chen, Jing Lv, Lei Hou, Zhao Dong, Rongfei Wang, Min Su, Shengyuan Yu

**Affiliations:** 1 Reward, Competition and Social Neuroscience Lab, Department of Psychology, School of Social and Behavioral Sciences, Nanjing University, Nanjing, China; 2 Institute for Brain Sciences, Nanjing University, Nanjing, China; 3 Medical School of Chinese PLA, Beijing, China; 4 Department of Neurology, the first Medical Center, Chinese PLA General Hospital, Fuxing Road 28, Haidian District, Beijing, 100853 China; 5 Department of Psychiatry, The 967th Hospital of Joint Logistic Support Force of PLA, Dalian, China; 6 Department of Medical Psychology, The Second Medical Center, Chinese PLA General Hospital, Beijing, China

**Keywords:** Microstate, Resting-state, Migraine, MwoA, EEG

## Abstract

**Background:**

Resting-state EEG microstates are thought to reflect brief activations of several interacting components of resting-state brain networks. Surprisingly, we still know little about the role of these microstates in migraine. In the present study, we attempted to address this issue by examining EEG microstates in patients with migraine without aura (MwoA) during the interictal period and comparing them with those of a group of healthy controls (HC).

**Methods:**

Resting-state EEG was recorded in 61 MwoA patients (50 females) and 66 HC (50 females). Microstate parameters were compared between the two groups. We computed four widely identified canonical microstate classes A-D.

**Results:**

Microstate classes B and D displayed higher time coverage and occurrence in the MwoA patient group than in the HC group, while microstate class C exhibited significantly lower time coverage and occurrence in the MwoA patient group. Meanwhile, the mean duration of microstate class C was significantly shorter in the MwoA patient group than in the HC group. Moreover, among the MwoA patient group, the duration of microstate class C correlated negatively with clinical measures of headache-related disability as assessed by the six-item Headache Impact Test (HIT-6). Finally, microstate syntax analysis showed significant differences in transition probabilities between the two groups, primarily involving microstate classes B, C, and D.

**Conclusions:**

By exploring EEG microstate characteristics at baseline we were able to explore the neurobiological mechanisms underlying altered cortical excitability and aberrant sensory, affective, and cognitive processing, thus deepening our understanding of migraine pathophysiology.

## Introduction

Migraine is characterized by recurrent headache attacks which exert a significant impact on the daily lives of sufferers [1, 2]. Although our understanding of the exact pathogenetic mechanisms behind migraine remains incomplete, it is now widely accepted that migraine, at its core, represents a complex brain network disorder [3, 4]. Over the last decade, we have witnessed remarkable progress in understanding the causes of migraine. Such progress can, to a large degree, be ascribed to an increased effort into examining the neural function of the migraine brain. Among others, measuring neural function with electrophysiological methods (i.e., Electroencephalogram, EEG) has been demonstrated to be an effective approach in describing migraine pathophysiology [5–8].

Within this domain, findings regarding migraine pathophysiology were primarily derived from task-oriented EEG studies that sought to determine electrophysiological activity associated with the performance of an explicit task [5]. Such studies have provided compelling evidence showing that migraine is associated with a state of functional cortical disexcitability, usually reflected by abnormal cortical evoked responses, across sensory, affective, and cognitive processes [9–11]. Despite these encouraging results, this type of task-oriented EEG is unable to fully capture all aspects of migraine pathophysiology. Migraine-related neural abnormalities at rest represent a clear example of the limitations of such a method [12]. For this reason, over the past decade, we have witnessed a surge in interest in the use of resting-state EEG, in which migraineurs refrain explicit activity and usually keep their eyes closed, to evaluate neural abnormalities that cannot be identified using task-oriented EEG [13]. The majority of previous resting-state EEG studies have focused on dynamic changes in spectral patterns and functional connectivity networks in migraineurs [14–20]. This conventional resting-state EEG analysis relied primarily on the examination of power- or oscillation-related variation in different frequency bands that integrate brain activity over seconds. Although these studies have revealed some new insights into migraine-related neural abnormalities, such a traditional analysis is not capable of detecting spatial and temporal properties of resting-state brain networks that occur on shorter time scales (e.g., within fractions of seconds).

Compared to the traditional resting-state EEG analysis, by using multichannel EEG on a sub-second time scale, EEG microstate analysis offers the promise of capturing the spatiotemporal dynamics of several components of resting-state brain networks at the whole-brain level [21, 22]. EEG microstates are often referred to as global patterns of spatial configurations of electric potentials that dynamically evolve over time in an organized manner [23]. Two key properties of EEG microstates have consistently been identified across studies [24]. First, although there are a large number of possible spatial configurations, they can typically be classified into four canonical classes, labeled A, B, C and D. These four classes typically explain 65–84% of total topographic variance [22]. Second, a single configuration is a brief period (about 60–120 ms) in which its spatial configuration remains dominant and quasi-stable before rapidly transitioning to another configuration. These periods of quasi-stability of a single configuration are thus called “microstates”. These key properties may serve EEG microstates well in the detection of alterations in rapid, dynamic activity in large-scale resting-state brain networks in neuropsychiatric disorders. Over the past decade, an increasing number of resting-state EEG studies have demonstrated the potential utility of a set of EEG microstate parameters (duration, occurrence, time coverage, and syntax) in detecting neurophysiological changes underlying certain neuropsychiatric disorders [21, 22]. To date, previous research has identified alterations in certain microstate parameters in neuropsychiatric conditions such as schizophrenia [25], major depressive disorder [26], panic disorder [27], autism spectrum disorder [28, 29], Alzheimer’s disease [30] and Parkinson’s disease [31]. Overall, previous work clearly suggests an intriguing relationship between features of certain EEG microstates and the neurophysiological basis of these neuropsychiatric disorders. These findings can in turn provide us with novel insights into the pathophysiological mechanisms underlying such disorders.

Surprisingly, our current understanding of the characteristics of resting-state EEG microstates in migraineurs is still lacking. To this end, we evaluated EEG microstates in MwoA patients during the interictal period, the period between migraine attacks, in comparison with a group of HC participants. Based on previous brain imaging studies reporting aberrant resting-state brain networks associated with migraine [32–34], we expected to observe changes in certain microstate parameters in the MwoA patient group compared to the HC group. Specifically, previous brain imaging studies have found a functional impairment in the form of visual cortex and attentional network hyperexcitability in migraineurs [35–38]. Given that microstate classes B and D have been shown to be linked to activities in the visual network (VN) and in the dorsal attention network (DAN) respectively [39], we can expect an increased presence of these two microstate classes in the MwoA patient group compared to the HC group. Meanwhile, a functional impairment in the form of the salience network (SN) disexcitability in migraineurs has also been observed [40]. Since microstate class C has been shown to be linked to activities in the SN [39], we can expect a reduced presence of microstate class C in the MwoA patient group compared to the HC group.

## Methods and materials

### Participants

We based the participant recruitment procedure on our recent study [10]. The patient group comprised of 61 MwoA patients (age = 32.79 ± 6.83, 50 females), all of whom had received diagnoses by trained neurologists (Z.D. and S.Y.) as well as neuropsychologists (G.C. and J.L.). Each patient kept a headache diary and completed structured questionnaires on demographics, headache profile, medical history, and medication use. The headache profile examined migraine history (years), the frequency (amount per month) and duration (days per month) of headaches, the severity of migraines, and included the six-item Headache Impact Test (HIT)-6, Hamilton Anxiety Scale (HAMA) and Hamilton Depression Scale (HAMD). The inclusion criteria for MwoA patients were: 1) fulfilling the diagnosed criteria for migraine according to the International Classification of Headache Disorders, 3rd edition (ICHD-3) and 2) a history of at least 2 years of migraine and at least one migraine episode per month. Moreover, individuals were to be excluded according to the following criteria: 1) neurological diseases (i.e., epilepsy, cerebral infarction, encephalitis, neuromuscular disorders); 2) mental retardation; 3) a current or past history of substance dependence; 4) receiving prophylactic anti-migraine therapy; 5) depressive and anxiety disorders (scores more than 7 points in HAMA and HAMD). We also employed an age- and sex-matched healthy control group consisting of 66 healthy volunteers (age = 31.44 ± 4.63, 50 females), none of whom reported any personal or family history of psychiatric or neurological disorders. This was confirmed by both a self-reported past history and a psychiatric examination of present mental state using the DSM-IV criteria of axis I. None of the female participants from either group took any oral contraceptives for at least 1 week prior to involvement in this study. All participants in the two groups were right-handed and signed informed consent forms. The study protocol was approved by the Ethics Committee of the Chinese PLA General Hospital. Demographic and clinical characteristics are described in Table 1.

**Table 1:** Demographic and clinical characteristics of the study sample

	MwoA patients (***n*** = 61)	HC (***n*** = 66)	Group comparison
	(M ± SD)	(M ± SD)	
Age, years	32.79 ± 6.83	31.44 ± 4.63	t(125) = − 1.31, *p* = 0.20
Gender (F/M)	(50/11)	(50/16)	χ^2^ = 0.41, *p* = 0.52
Education, years	15.76 ± 3.16	15.87 ± 3.08	t(125) = − 0.20, *p* = 0.84
BMI [kg/m^2^]	21.56 ± 3.02	21.81 ± 2.74	t(125) = 0.50, *p* = 0.62
MoCA	28.18 ± 1.12	28.50 ± 0.90	t(125) = 1.78, *p* = 0.08
Duration of migraine, days per month	4.38 ± 3.12		
History of migraine, years	11.38 ± 6.47		
Migraine frequency, times per month	3.15 ± 1.74		
Severity of headache (VAS scale)	7.72 ± 1.55		
HIT-6	65.85 ± 6.91		

*VAS* visual analog scale, with 0 indicating no pain and 10 worst possible pain, *BMI* body mass index, *MoCA* the Montreal Cognitive Assessment, *HIT-6* the six-item Headache Impact Test, *M* mean, *SD* standard deviation, *HC* healthy controls, *MwoA* migraine without aura

### EEG data acquisition and preprocessing

We employed an EEG data recording procedure similar to that described in our previous studies [10, 41, 42]. Resting-state EEG data were recorded (SynAmps amplifier, NeuroScan) with a quick cap carrying 64 Ag/AgCl electrodes placed at standard locations covering the whole scalp (the extended international 10–20 system). The reference electrode was attached to the right mastoid (M2), and the ground electrode was placed on the forehead. The vertical electrooculogram (VEOG) was recorded with electrodes placed above and below the left eye. The horizontal electrooculogram (HEOG) was recorded using electrodes placed beside the two eyes. Impedance was kept below 5 kΩ. Electrophysiological data were continuously recorded with a bandwidth of 0.05–100 Hz and sampled at a rate of 1000 Hz. All participants were asked to keep their eyes closed and to relax throughout the recording period (4 min).

Offline EEG data were down-sampled to 500 Hz and preprocessed using EEGLAB 2021.0 [43]. Preprocessing analysis was consistent with the procedure reported in previous work [29, 44, 45]. Specifically, the raw EEG data were filtered with a bandpass of 0.5 – 70 Hz and a notch (50 Hz) filter. Upon visual inspection, epochs with artifacts caused by movement or poor signal were detected and removed manually. Independent component analysis (ICA) was then used to remove eye movements-, muscular- and bad channel-related artifacts. After EEG data preprocessing, 60 artifact-free epochs of 2 s duration in each group were selected for analysis. Finally, data were re-referenced to a common average reference.

### EEG microstate analysis

Consistent with recent work [44], we performed microstate analysis using the Microstate Analysis plug-in (Version 1.1) for EEGLAB [43]. This adhered to well-established standard procedures reported in previous studies [46, 47]. Briefly, preprocessed EEG data were digitally filtered with a bandpass of 2–20 Hz. Then, we computed the Global Field Power (GFP) for each participant, which represents the overall potential variance across all electrodes at each sample in time. Given that EEG scalp topographies around the peaks of the GFP remain stable, we only extracted and submitted topographies at the momentary peaks of GFP to subsequent analysis. Four classes of microstate topography have previously been found to optimally account for EEG data variance and have frequently been adopted in the existing research on neuropsychiatric disorders [22]. We thus computed four microstate class topographies in the present study using a modified version of the K-mean clustering algorithm [48]. Microstate class topographies for each group were calculated separately using a permutation algorithm that minimized common variance across participants [49]. We labeled the four microstate classes as A, B, C, or D according to their similarities to the microstate class topographies reported in previous work [21]. For each participant, four microstate parameters were computed for each class: mean duration (ms) (the average time that a given microstate was continuously present), occurrence (the mean number of a given microstate per second), time coverage (%) (the percentage of total analysis time spent in a given microstate) and syntax (the transition from each of the four microstate classes to any other microstate classes). Additionally, microstate class topographies between the two groups were compared using a topographic analysis of variance (TANOVA) [50], as implemented in the Ragu software [51].

### Statistical analysis

We used a non-parametric chi-square test to assess group differences in gender ratio. Independent sample t-tests were employed to examine between-group differences in age and body mass index (BMI). To assess between-group differences in microstate parameters (mean duration, occurrence and time coverage), we ran three separate mixed analyses of variance (ANOVA), with group as a between-participants factor (MwoA patients versus HC) and microstate class (A versus B versus C versus D) as a within-participants factor. Regarding microstate syntax analysis, we followed a procedure described in previous work [26, 45, 52, 53], in which we computed the percentage of transitions from one microstate class to another. We achieved this by calculating the relative observed occurrence of transitions from one microstate class to all other classes. After normalization, we obtained the percentage for each possible transition for every participant in this study. Subsequently, while Bonferroni correcting for multiple comparisons, we performed a two-sample t-test on each pair of microstate class transitions to examine whether there were significant between-group differences in the transition probabilities. Finally, for microstates showing significant between-group differences, we performed Pearson’s correlations to assess the relationships between microstate parameters and clinical measures.

All data were analyzed using R (version 4.1.0). Statistical comparisons were made at *p*-values of *p* < .05, with the Greenhouse–Geisser correction when violations of sphericity occurred.

## Results

### Microstate maps

**Fig. 1:**
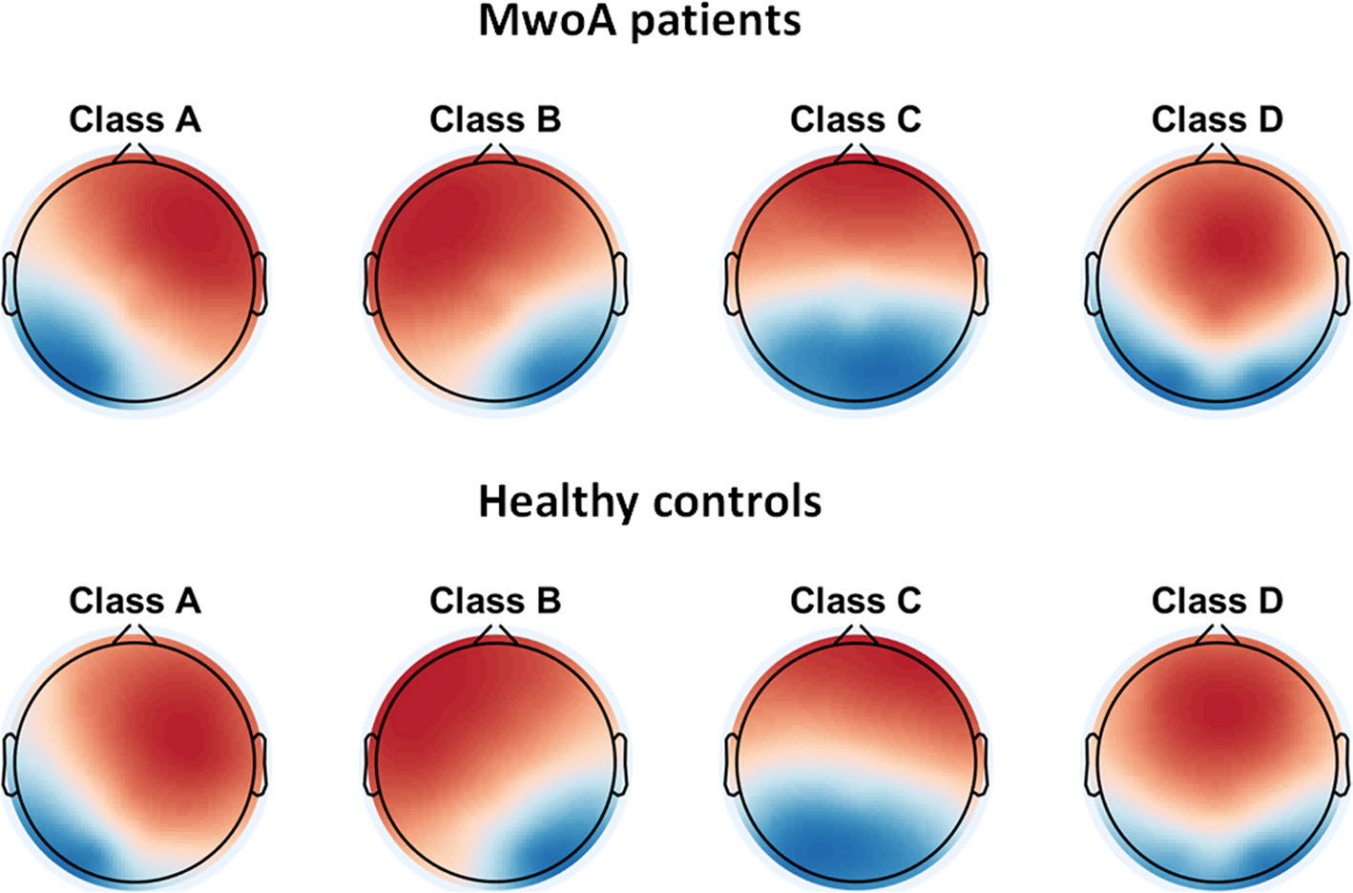
The spatial configuration of the four microstate classes, separately for MwoA patients and healthy controls. Each row shows the four topographic configurations (A-D) for each group. MwoA, migraine without aura

The topographies of 4 dominant microstate classes strongly resembled those reported in previous work [21, 22, 45, 52]. These four microstate classes explained more than 77% of the global variance in each group (79.58% in the MwoA patient group and 77.45% in the HC group) (Fig. 1). Therefore, we were able to categorize them as microstate classes A, B, C, and D. Additionally, the topographic analysis of variance (TANOVA) analysis on microstate class topographies between the two groups did not reveal either a significant main effect of group (*p* = 0.456) or a significant group × microstate class interaction (*p* = 0.932), demonstrating that there were no significant differences in microstate class topographies between the two groups.

### Mean duration (ms)

**Fig. 2:**
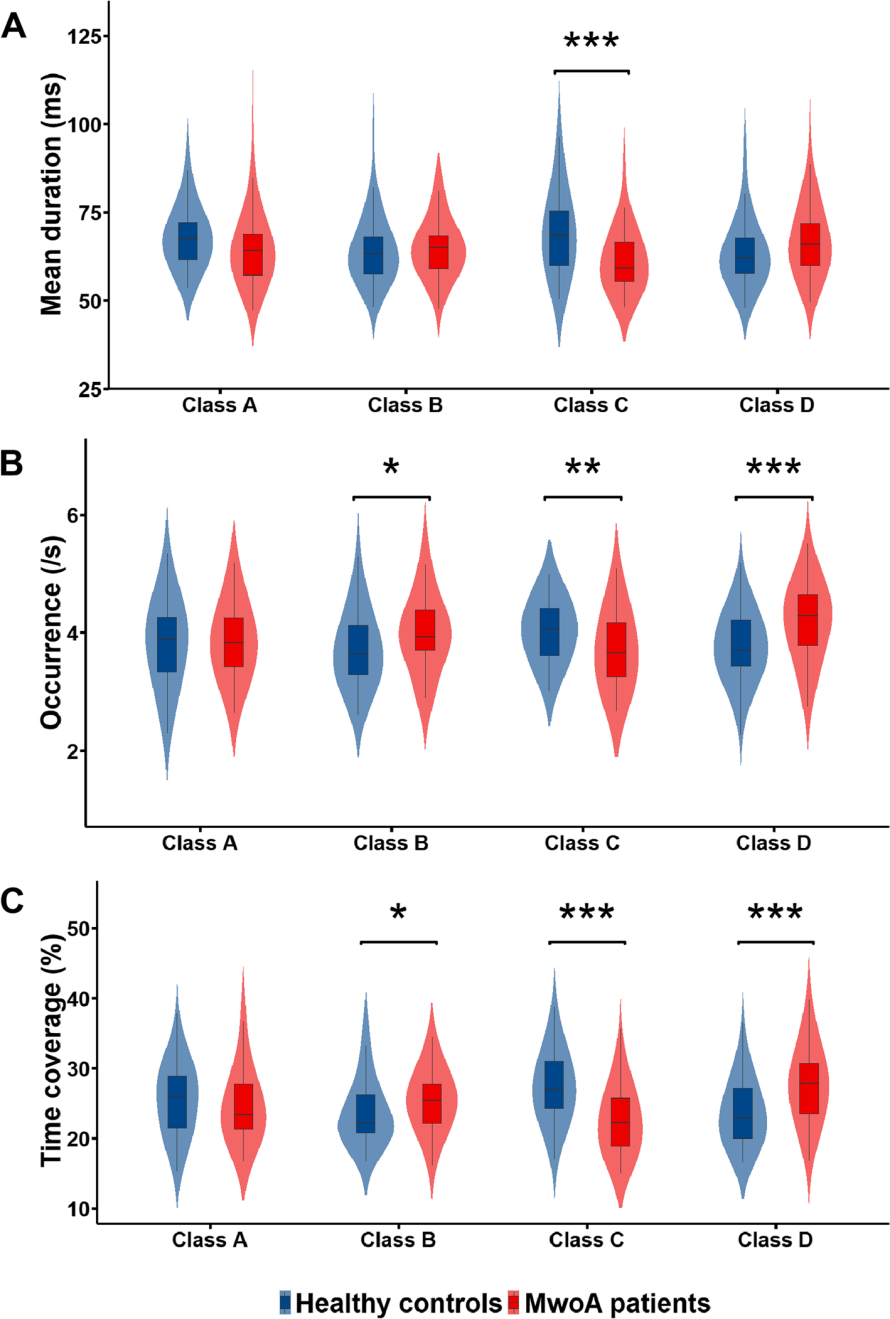
Microstate analysis of temporal parameter results. **A** Violin plots showing the mean duration of each microstate class in each of the two groups. The decreased duration of microstate class C was found in the MwoA patient group compared to the HC group; **B** Violin plots showing the occurence of each microstate class in each of the two groups. The increased occurence of microstate classes B and D, but decreased occurence of microstate class C, were found in the MwoA patient group compared to the HC group; **C** Violin plots showing the time coverage of each microstate class in each of the two groups. The increased time coverage of microstate classes B and D, but decreased time coverage of microstate class C, were found in the MwoA patient group compared to the HC group. MwoA, migraine without aura; HC, healthy controls; *p < .05, **p < .01, ***p < .001

A mixed ANOVA did not reveal a significant main effect of either group (F(1, 125) = 1.72, *p* = 0.19) or microstate class (F(3, 375) = 1.33, *p* = 0.26). However, we found a significant group × microstate class interaction (F(3, 375) = 15.49, *p* < .001). An analysis of simple effects revealed that the mean duration of microstate class C was shorter in the MwoA patient group than in the HC group (*p* < .001) (Table 2, Fig. 2A).

**Table 2:** MwoA patients vs. healthy controls for all microstate parameters and for each microstate class

Microstate	MwoA patients (M ± SD)	HC (M ± SD)
**Mean duration (ms)**		
Class A	65.13 ± 10.94	68.06 ± 8.50
Class B	64.99 ± 8.23	64.74 ± 9.67
Class C	61.44 ± 8.70	69.45 ± 11.52
Class D	63.61 ± 10.03	67.25 ± 9.68
**Occurrence (/s)**		
Class A	3.86 ± 0.62	3.83 ± 0.70
Class B	3.99 ± 0.67	3.72 ± 0.62
Class C	3.70 ± 0.65	4.04 ± 0.51
Class D	4.18 ± 0.62	3.76 ± 0.55
**Time coverage (%)**		
Class A	24.55 ± 5.18	25.51 ± 4.81
Class B	25.41 ± 4.11	23.64 ± 4.52
Class C	22.41 ± 4.62	27.30 ± 4.82
Class D	27.62 ± 5.15	23.56 ± 4.45

*M* mean, *SD* standard deviation, *HC* healthy controls, *MwoA* migraine without aura

### Occurrence (times/S)

Using a mixed ANOVA, we found no significant main effects of either group (F(1, 125) = 1.65, *p* = 0.20) or microstate class (F(3, 375) = 1.64, *p* = 0.18). However, there was a significant interaction between group and microstate class (F(3, 375) = 12.58, *p* < .001). An analysis of simple effects revealed that microstate classes B (*p* < .05) and D (*p* < .001) were significantly more frequent in the MwoA patient group than in the HC group, while the microstate class C was significantly less frequent in the MwoA patient group than in the HC group (*p* < .01) (Table 2, Fig. 2B).

### Time coverage (%)

A mixed ANOVA failed to reveal significant main effects for either group (F(1, 125) = 1.02, *p* = 0.32) or microstate class (F(3, 375) = 0.85, *p* = 0.46). However, we did find a significant interaction between group and microstate class (F(3, 375) = 15.84, *p* < .001). An analysis of simple effects revealed that microstate classes B (*p* < .05) and D (*p* < .001) covered significantly more time in the MwoA patient group than in the HC group, while microstate class C covered significantly less time in the MwoA patient group than in the HC group (*p* < .001) (Table 2, Fig. 2C).

### Microstate syntax

**Fig. 3:**
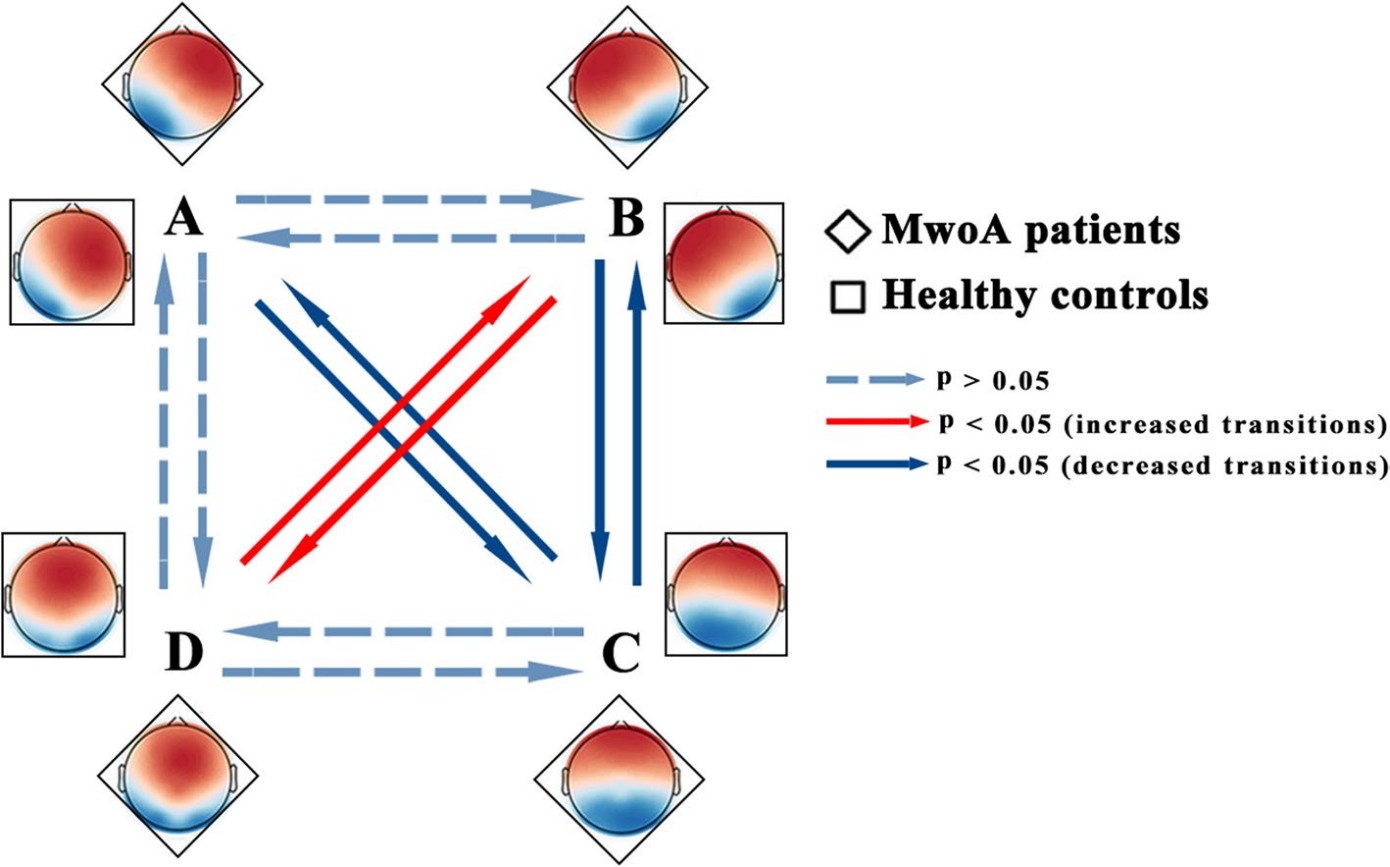
Schematic view of microstate syntax analysis results. A Significant differences in transition probabilities for each pair of microstate class between MwoA patients and healthy controls were found. The MwoA patient group had a bias toward fewer transitions from A to C, B to C, C to A and C to B than the HC group. In contrast, the MwoA patient group had a bias toward more transitions from B to D and D to B than the HC group. MwoA, migraine without aura; HC, healthy controls

The percentage of observed transitions from one microstate class to all other classes in the MwoA patient and HC groups is shown in Table 3. We performed t-tests to examine observed transition probabilities between groups and found that, compared to the HC group, the MwoA patient group showed a bias toward making the fewer transition from one microstate to another. Specifically, we observed this bias between the following microstates: A to C (t(125) = 4.81, *p* < .001); from B to C (t(125) = 3.39, *p* < .05); from C to A (t(125) = 5.23, *p* < .001) and from C to B (t(125) = 3.54, *p* < .01) (Fig. 3). In contrast, we found that the MwoA patient group showed a bias toward making more transitions from B to D (t(125) = − 4.69, *p* < .001) and from D to B (t(125) = − 5.64, *p* < .001) than the HC group (Fig. 3).

**Table 3:** Percentage of transitions from one microstate class to all other classes in MwoA patients and healthy controls

Transition	MwoA patients (M ± SD)	HC (M ± SD)
A to B	7.92 ± 1.74	7.42 ± 1.79
A to C	7.00 ± 1.82	8.58 ± 1.87
A to D	8.67 ± 1.81	7.92 ± 2.04
B to A	8.06 ± 1.76	7.33 ± 1.78
B to C	7.38 ± 1.84	8.54 ± 1.97
B to D	8.88 ± 2.14	7.29 ± 1.65
C to A	6.99 ± 1.74	8.71 ± 1.93
C to B	7.30 ± 1.84	8.51 ± 1.98
C to D	8.24 ± 1.70	8.29 ± 1.78
D to A	8.53 ± 1.89	7.88 ± 2.05
D to B	9.14 ± 2.17	7.34 ± 1.36
D to C	8.15 ± 1.79	8.36 ± 1.92

*M* mean, *SD* standard deviation, *HC* healthy controls, *MwoA* migraine without aura

### Correlation between microstate parameters and clinical measures

**Fig. 4:**
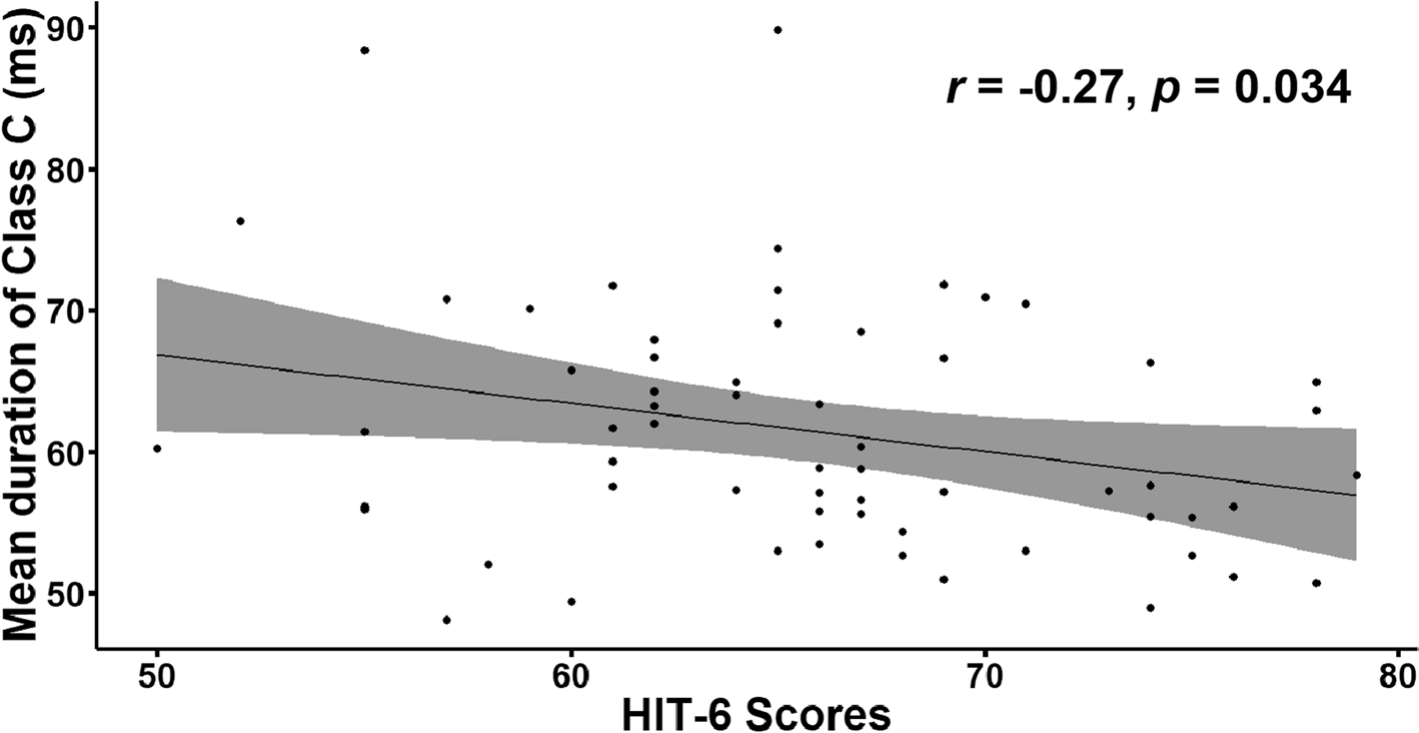
Microstate class C association with clinical measures. Scatterplot of mean duration of microstate class C and the six-item Headache Impact Test (HIT-6) scores in MwoA patients. MwoA, migraine without aura

Our correlation analysis only revealed a significantly negative correlation between the mean duration of microstate class C and HIT-6 scores in the MwoA patient group (*r* = − 0.27, *p* < .05) (Fig. 4).

## Discussion

The present study demonstrates the presence of abnormalities in resting-state EEG microstates among MwoA patients. Overall, MwoA patients exhibited divergent temporal microstate profiles compared to those in the HC group. Moreover, the MwoA patient group, relative to the HC group, displayed multiple distinct microstate transition probabilities, which primarily involved microstate classes B, C, and D. Here, we will discuss how these findings may help to shed light on migraine pathophysiology.

### Microstate class B and the Visual Network (VN)

We found that microstate class B displayed higher time coverage and occurrence in the MwoA patient group than in the HC group. Microstate class B has been shown to be linked to activities in the VN encompassing the bilateral lateral extrastriate visual areas [39]. A clear change in this microstate class may thus represent underlying structural abnormalities in this area in MwoA patients. This argument is supported by previous anatomical imaging studies reporting anatomical alterations in the VN (e.g., increased cortical thickness) in MwoA patients [54]. Consistent with the notion that brain areas showing structural abnormalities in migraineurs also show functional alterations, structural alterations in the VN may provide an explanation for the functional changes that we observe in microstate class B.

Meanwhile, such a change in microstate class B may also indicate functional alterations of the VN in MwoA patients at baseline. That is, these results reflect activity occurring during default functional states that are independent of any task performance. Occurrence and time coverage of a particular microstate class has usually been interpreted to reflect the tendency of its underlying cortical and subcortical sources to be activated as well as the corresponding relative time coverage of such underlying neural activities [22]. It is thus reasonable to argue that an increase in these parameters for microstate class B at rest in MwoA patients would produce the same implications. That is an enhanced tendency of its underlying cortical and subcortical sources to be activated as well as an increase in the corresponding relative time coverage of such underlying neural activities, indicating an enhanced likelihood of neural activation of the VN in response to visual events. This speculation is indeed supported by previous functional studies in migraineurs which have converged to reveal a functional impairment in the form of visual cortex hyperexcitability in migraineurs [55–57]. In this sense, our observation of increased microstate class B activity at rest may provide an insightful clue regarding how rapidly fluctuating microstates at rest may contribute to the visual disturbances and visual hyperactivity in the visual system of MwoA patients.

It should be noted that patients in the present study all suffered from migraines without aura. The effect observed in these patients adds to a growing literature showing that visual hyperactivity in the visual system can also be found in MwoA patients [35, 58, 59]. In spite of some ongoing controversy [36] and their differing clinical symptoms [60], our finding provides adds support to the viewpoint that similar pathogenic mechanisms may be shared among all migraine patients, both with visual aura (usually with coexisting visual disturbances) and without aura.

### Microstate class C and the Salience Network (SN)

In contrast to microstate class B, we found a significant decrease in microstate class C (mean duration, occurrence, and time coverage) in the MwoA patient group compared to the HC group. This microstate class has been related to the salience network (SN) focusing mainly on the dorsal anterior cingulate cortex (dACC) and anterior insula (AI) [39]. Decreased microstate class C may be associated with the structural abnormalities in the SN that have been reported by previous anatomical imaging studies [61]. These three microstate parameters (mean duration, occurrence, and time coverage) have been interpreted to reflect three underlying neurophysiological mechanisms: the average length of time a given microstate class remains stable; the tendency of its underlying cortical and subcortical sources to be activated; and the corresponding relative time coverage of such underlying neural activities [22]. Thus, a decrease in these three parameters in microstate class C indicates the following: a reduction in the average length of time a given microstate class remains stable; a reduction in the tendency of its underlying cortical and subcortical sources to be activated; and a reduction in the corresponding relative time coverage of such underlying neural activities. Such indications thereby suggest a functional impairment of the SN in MwoA patients at baseline. This would be consistent with previous resting-state fMRI studies showing reduced intrinsic connectivity within the SN in these patients [40].

Regarding the functional significance of the SN, several views have emerged to provide a possible explanation of its activity during resting state. A prevailing viewpoint to the well-established role of the SN in interceptive awareness and sensory processing of salient events [62, 63]. This view emphasizes its role in detecting and filtering salient stimuli and in coordinating other brain networks (e.g., central-executive network (CEN)) to guide behavior. In addition to this view, recent studies have begun to identify a specific role of this network in inhibitory control [64, 65]. Despite some controversy, one line of evidence supports the notion that the SN integrates salient information that is subsequently used by the CEN, including the inferior frontal cortex (IFC), for recruiting inhibition [65, 66]. This implies an indirect involvement of salience processing in inhibitory control. Therefore, we can speculate that dysfunctional SN may lead to an aberrant assignment of salience to sensory stimuli. This is then improperly, or incompletely, processed by the CEN and ultimately causes reduced involvement of the CEN in recruiting inhibition in MwoA patients. From this perspective, it is possible that the aberrant role of salience processing in inhibitory control in MwoA patients is related to hypervigilance to salient events (e.g., ongoing pain and sensory stimuli), which are common triggers for migraine headaches. This argument is partly supported by aberrant syntax patterns in the MwoA patient group compared to the HC group observed in the present study. A reduction in transitions from microstate class C to microstate class B and A seems to implicate decreased functional connectivity from the SN to the primary sensory networks. Such a pattern may then lead to an increase in cortical excitability and sensory gain, as implied by our observation of increased microstate class B in association with more engagement of the VN in MwoA patients. This finding is in agreement with previous work showing decreased SN and CEN connectivity [40] and decreased SN and VN connectivity in migraineurs [67–69]. More importantly, these findings further indicate that the SN may stand at a ‘crossroads’ in the network architecture of the migraine brain and consequently may represent a potential target for improving the adverse impact of headache on daily functions in sufferers [67]. Our observation of negative associations in the MwoA patient group between the mean duration of microstate class C and Hit-6 scores, which measure the adverse impact of headache on social functioning, role functioning, vitality, cognitive functioning and psychological distress, appears to support this argument.

### Microstate class D and the dorsal attention network (DAN)

Finally, we observed a significant increase in time coverage and occurrence of the microstate class D in the MwoA patient group compared to the HC group. In accordance with previous work [39], this microstate class is related to activities in the DAN including the dorsal areas of the frontal and parietal cortex. Our finding thus implies a functional impairment in this network among MwoA patients. Increases in these two parameters for microstate class D at baseline seem to suggest potential hyperexcitability of the DAN to incoming sensory stimuli. This is consistent with recent brain imaging studies showing an increase in neural responses to both attended and unattended stimuli in the key regions of the DAN [37, 38]. Furthermore, the functional significance of the DAN has been argued to reflect reflexive aspects of attention, such as switching and reorientation of attention to relevant information [70]. From this, we can speculate that migraineurs may exhibit an exaggerated pattern of reflexive orienting responses to incoming sensory stimuli. This argument is indeed supported by previous work showing heightened reflexive visual-spatial orienting to attended and unattended events [71–73]. In this sense, the change in microstate class D at baseline observed in MwoA patients would thus be associated with alterations in top-down and/or bottom-up attention during task performance. Such an interpretation is supported by our observation of aberrant syntax patterns in the MwoA patient group compared to the HC group. Here, we observed increased transitions from microstate class D to microstate class B, a finding that is also consistent with previous resting-state studies showing increased functional connectivity between the DAN and VN [68, 74]. Thus, it is possible that such atypical syntax patterns in the MwoA patient group at baseline may provide a potential neurobiological explanation for the enhanced attentional focus toward visual events described above.

### Potential limitations

Despite the relatively large sample size used in the present study, we should consider several potential limitations. First, only MwoA patients were involved in the present study. Thus, it remains unclear as to whether these findings can be generalized to other types of migraine groups, such as patients suffering from migraine with aura (MA) and chronic migraine patients. It would be important to address this issue in future studies mainly because different pathophysiological mechanisms have been found to play a role in these different types of headache syndromes [75, 76]. Second, the present study is not capable of allowing us to identify whether deviant temporal microstate profiles found in MwoA patients represent the trait or state nature of microstate abnormalities. Taking this into account in future studies would further help to shed additional light on migraine pathophysiology. Third, in recent years, we have witnessed an increased effort to evaluate the reliability of EEG microstate analysis. Current literature shows that EEG microstate results remain highly stable, independent of the methods used to determine the cluster maps [24, 77] the number of recording electrodes [24, 78], the duration of the epoch [79], and EEG reference-dependent problems [80]. However, other factors, which remain to be systematically investigated, may influence the reliability of EEG microstate analysis. For this reason, we can say that the direct examination of the cortical source maps of each microstate class would have the added benefit of proving the relationship between each microstate class and its underlying resting-state networks.

## Conclusion

In sum, in this study we showed divergent temporal microstate profiles and aberrant microstate syntax patterns in the MwoA group compared to the HC group. The divergent temporal microstate profiles in the MwoA patient group reflect an increase in baseline brain activities in the VN and the DAN, alongside a reduction in neural activities in the SN at baseline. Moreover, we were also able to observe a decrease in transitions from the SN to the VN as well as an increase in transitions from the DAN and the VN in MwoA patients. This is consistent with previous research demonstrating aberrant functional connectivity among diversely distributed resting-state brain networks in migraineurs. Meanwhile, it is worth noting that EEG microstate analysis offers an important and complementary approach to the detection of large-scale resting-state networks in the migraine brain as performed in many resting-state fMRI studies. Specifically, by exploiting the high temporal information inherent in resting-state EEG signals and capturing rapidly fluctuating microstates [21], EEG microstate analysis can provide information regarding resting-state networks in the migraine brain distinct from resting-state fMRI measures focused solely on slowly oscillating resting states. Compared with resting-state fMRI analysis, EEG microstate analysis is also more efficient in terms of its ability to inexpensively test large groups of participants. In this sense, by providing distinct yet complementary information while circumventing some of the inherent limitations of other brain imaging techniques (e.g., functional MRI), this approach can advance our understanding of migraine pathophysiology. Taken together, these findings may expand our understanding of the altered cortical excitability, enhanced attentional focus toward sensory events, and enhanced sensory gain that is present in migraineurs.

## Data Availability

The datasets used and analyzed during the present study are available from the corresponding authors on reasonable request.

## Abbreviations

MwoA: Migraine without aura
HC: Healthy controls
MA: Migraine with aura
EEG: Electroencephalogram
VN: Visual network
SN: Salience network
DAN: Dorsal attention network
HIT: Headache Impact Test
CEN: Central-executive network
IFC: Inferior frontal cortex
dACC: Dorsal anterior cingulate cortex
BMI: Body mass index
ANOVA: Analyses of variance
GFP: Global Field Power
VEOG: Vertical electrooculogram
HEOG: Horizontal electrooculogram
HAMA: Hamilton Anxiety Scale
HAMD: Hamilton Depression Scale
ICHD: International Classification of Headache Disorders
